# Dysphagia Phenotypes in COVID-19 Pneumonia Versus Aspiration Pneumonia: A Retrospective Quantitative Videofluoroscopic Cohort Study

**DOI:** 10.3390/medicina62071212

**Published:** 2026-06-23

**Authors:** Su Jung Park, Hyun Seok, Sang-Hyun Kim, Seung Yeol Lee, Beom Jin Kim, Taehwan Park, Eunho Kim, Hyun Jung Kim

**Affiliations:** Department of Physical Medicine and Rehabilitation, Soonchunhyang University Bucheon Hospital, Soonchunhyang University College of Medicine, 170 Jomaru-ro, Wonmi-gu, Bucheon 14584, Gyeonggi-do, Republic of Korea; iawpsj94@gmail.com (S.J.P.);

**Keywords:** COVID-19 pneumonia, aspiration pneumonia, dysphagia, videofluoroscopic swallowing study, pharyngeal residue, Normalized Residue Ratio Scale, upper esophageal sphincter, epiglottic inversion

## Abstract

*Background and Objectives:* Comparisons of swallowing physiology between coronavirus disease 2019 (COVID-19) pneumonia and clinically diagnosed aspiration pneumonia (AP) have largely relied on ordinal scales, leaving etiology-specific biomechanical profiles unclear. We quantitatively compared videofluoroscopic swallowing study (VFSS) measures of pharyngeal residue and clearance mechanics to identify differential dysphagia phenotypes. *Materials and Methods:* This single-center retrospective cohort study included 50 adult inpatients with pneumonia (COVID-19, *n* = 25; AP, *n* = 25) who underwent VFSS for suspected dysphagia. COVID-19 pneumonia was laboratory-confirmed, and AP was clinically diagnosed after negative tests for severe acute respiratory syndrome coronavirus 2. Blinded ImageJ analysis examined the first standardized semisolid yogurt swallow (International Dysphagia Diet Standardisation Initiative level 4). Primary outcomes were Normalized Residue Ratio Scale values for the valleculae (NRRSv) and piriform sinuses (NRRSp); secondary outcomes were upper esophageal sphincter (UES) opening width and epiglottic rotation angle. Penetration–Aspiration Scale (PAS) score, hyoid displacement, and pharyngeal transit time were exploratory. *Results:* Baseline characteristics were comparable. COVID-19 pneumonia showed higher NRRSv (0.20 [0.12–0.56] vs. 0.13 [0.00–0.20]; *p* = 0.01). NRRSp was numerically higher but not statistically significant (0.12 [0.00–0.43] vs. 0.00 [0.00–0.17]; *p* = 0.07). COVID-19 pneumonia also showed smaller UES opening width (5.08 ± 2.48 vs. 6.50 ± 2.01 mm; *p* = 0.03) and reduced epiglottic rotation angle (66.0 [29.0–80.8] vs. 93.4 [74.2–100.4] degrees; *p* = 0.04). No statistically significant between-group difference was detected in PAS-defined airway invasion severity on the standardized semisolid task. *Conclusions:* These findings suggest an efficiency-predominant dysphagia phenotype in COVID-19 pneumonia, characterized by greater vallecular residue and restrictive clearance-related mechanics on a standardized semisolid task. The results indicate that PAS-defined safety metrics alone may underestimate residue-related dysphagia burden in this population.

## 1. Introduction

Swallowing is a complex sensorimotor process requiring the coordinated interplay of bolus propulsion and timely airway protection [1]. Disruption of these mechanics can lead to dysphagia [1], commonly evident as post-swallow pharyngeal residue and airway invasion, with findings often interpreted as correlates of swallowing efficiency and swallowing safety, respectively [2]. Videofluoroscopic swallowing study (VFSS) enables dynamic assessment of these events [3], and the application of standardized quantitative metrics can reduce subjectivity [4] and facilitate phenotype-based characterization of dysphagia across different etiologies [5].

Aspiration pneumonia (AP) is a common infectious complication in frail older adults and in patients with chronic neurologic or systemic diseases [6]. Clinically diagnosed AP is typically conceptualized as a syndrome centered on aspiration or microaspiration risk; however, its underlying swallowing physiology is often heterogeneous because it arises from diverse risk factors and mechanisms [7]. As a result, although susceptibility to aspiration is a key clinical feature [7], aspiration severity may be variably captured on a single VFSS, and measures of swallowing efficiency may vary widely across individuals without yielding a consistent group-level physiological profile.

In contrast, dysphagia associated with coronavirus disease 2019 (COVID-19) pneumonia may plausibly reflect a more efficiency-predominant profile shaped by acute critical illness and its management [8]. In hospitalized patients, critical illness-related deconditioning and catabolic muscle loss, prolonged immobilization, and airway management-related laryngopharyngeal impairment can plausibly compromise the biomechanical drivers of pharyngeal clearance [9]. Such factors are expected to weaken bolus-driving forces and disrupt clearance mechanics, thereby increasing post-swallow residue [1]. In addition to critical illness-related deconditioning, potential neuromuscular and sensorimotor sequelae after severe acute respiratory syndrome coronavirus 2 (SARS-CoV-2) infection have been proposed as contributors to dysphagia. However, their direct relationship with VFSS-derived swallowing biomechanics remains incompletely defined [10,11]. Furthermore, as previous comparisons have largely relied on ordinal scales or limited timing measures [12,13], the specific videofluoroscopic quantitative differences in swallowing biomechanics and pharyngeal clearance physiology between hospitalized patients with COVID-19 pneumonia and those with clinically diagnosed AP remain uncharacterized.

Post-swallow residue is a clinically relevant marker of impaired bolus propulsion and pharyngeal clearance [14]. The Normalized Residue Ratio Scale (NRRS) provides an anatomically normalized, image-based quantification of residue in the vallecular and piriform sinus compartments, enabling standardized between-group comparisons [15]. To interpret residue mechanistically, quantitative measures linked to pharyngeal clearance are informative [16]. Restricted upper esophageal sphincter (UES) opening can impede hypopharyngeal outflow [17] and contribute to residue, particularly in the piriform sinuses [18]. Likewise, impaired epiglottic inversion, quantified by a reduced epiglottic rotation angle [19], may reflect restricted epiglottic mobility and less effective bolus clearance through the vallecular space, thereby predisposing to incomplete vallecular emptying and greater vallecular residue [20]. Additionally, hyoid displacement and pharyngeal transit time provide complementary kinematic and timing descriptors [3], while the Penetration–Aspiration Scale (PAS) characterizes airway invasion severity as a safety outcome [21].

Accordingly, we compared quantitative VFSS measures between hospitalized patients with COVID-19 pneumonia and those with clinically diagnosed AP who underwent VFSS for clinically suspected dysphagia, under a standardized semisolid bolus condition. We prespecified a primary hypothesis that the COVID-19 pneumonia group would demonstrate a greater burden of post-swallow residue than the AP group, operationalized as higher NRRS values in the valleculae and piriform sinuses. To assess residue within a clearance-focused physiological framework, we prespecified key secondary hypotheses that the COVID-19 group would exhibit more restrictive pharyngeal clearance mechanics, reflected by smaller UES opening width and a reduced epiglottic rotation angle. Hyoid displacement, pharyngeal transit time, and PAS outcomes were evaluated as exploratory kinematic, timing, and safety descriptors to further characterize the between-group swallowing profiles.

## 2. Materials and Methods

### 2.1. Study Design and Setting

This was a single-center retrospective cohort study conducted at the Soonchunhyang University Bucheon Hospital, a tertiary care academic hospital. We reviewed the medical records and VFSS videos of consecutive adult patients who were hospitalized with pneumonia between 1 January 2022 and 31 January 2023 and were referred to the Department of Rehabilitation Medicine for VFSS owing to suspected dysphagia. The study protocol was approved by the institutional review board (IRB) of the Soonchunhyang University Bucheon Hospital (IRB No. SCHBC 2025-12-001; approval date: 2 January 2026), which waived the requirement for individual informed consent because only de-identified data were analyzed. The study was performed in accordance with the Declaration of Helsinki.

### 2.2. Participants

For this study, pneumonia was operationally defined based on a clinical-radiologic definition, comprising compatible clinical symptoms (e.g., fever, cough, sputum production, dyspnea) together with new infiltrates on chest radiography or computed tomography [22]. Patients were allocated to one of two groups based on etiology and virological testing. The COVID-19 pneumonia group included patients who were hospitalized for pneumonia management and had laboratory-confirmed SARS-CoV-2 infection, defined as a positive real-time reverse-transcription polymerase chain reaction (RT-PCR) result from a nasopharyngeal or oropharyngeal swab [23]. To reduce etiologic misclassification, testing was restricted to specimens obtained within 2 weeks of symptom onset, an interval in which hospital-based SARS-CoV-2 nucleic acid amplification test has shown high diagnostic sensitivity [24]. The AP group included patients with two consecutive negative SARS-CoV-2 RT-PCR tests performed at presentation (within 2 weeks of symptom onset) and on repeat testing 24–48 h later [25], with no subsequent positive test during the index admission. The clinical diagnosis of aspiration pneumonia was made by the attending physicians and reviewed by the research team, based on compatible imaging patterns combined with at least one documented indicator of aspiration risk, including: (1) witnessed aspiration events or choking episodes; (2) aspiration or oropharyngeal pooling confirmed on prior instrumental assessments; or (3) a clinical history explicitly suggestive of aspiration [7]. In both groups, patients were referred for VFSS because dysphagia was clinically suspected. In this manuscript, AP reflects an operational clinical diagnosis and does not imply microbiological confirmation in all cases [7].

In both groups, we included adult patients (aged ≥18 years) who (1) had dysphagia symptoms prompting referral for VFSS; (2) were able to cooperate with the VFSS protocol [26]; (3) were hemodynamically stable with sufficient postural control to sit in a lateral position at the fluoroscopy unit [26]; and (4) had no known allergy to barium contrast. We excluded patients with a recent central nervous system (CNS) lesion (e.g., ischemic or hemorrhagic stroke or traumatic brain injury) that can directly affect swallowing function [27], with the lesion diagnosed within the preceding 6 months; those with a remote CNS lesion (>6 months before the index admission) who had a documented pre-existing history of dysphagia; those with progressive neurodegenerative or neuromuscular diseases known to cause dysphagia (e.g., Parkinson’s disease, amyotrophic lateral sclerosis) [27]; and those with structural lesions of the head and neck or upper esophagus (e.g., head and neck cancer, prior surgery or radiotherapy involving the pharynx or larynx, or esophageal stricture). Patients with incomplete VFSS data or fluoroscopic images of insufficient quality for quantitative analysis were also excluded.

The study size was determined by the number of eligible patients available during the prespecified retrospective study period. All consecutive eligible patients meeting the inclusion and exclusion criteria were included in the analysis. Since the cohort size was fixed by the available cases, no formal a priori sample size calculation was performed.

### 2.3. Demographic and Clinical Variables

Demographic and clinical background variables were extracted from the electronic medical records at the time of VFSS. These included age at VFSS (years), sex, cumulative durations (days) of endotracheal intubation and tracheostomy during the interval between pneumonia diagnosis and VFSS, total hospital days and total intensive care unit (ICU) days from pneumonia diagnosis to VFSS, and the time from pneumonia diagnosis to VFSS (days). These variables were collected to characterize the cohort and to explore potential between-group imbalances in illness severity and treatment course.

### 2.4. Videofluoroscopic Swallowing Study Protocol

VFSS was performed using our institutional standardized protocol in the lateral projection. Patients were seated upright and positioned next to the radiographic unit (AXIOM Luminos dRF; Siemens Healthineers, Erlangen, Germany). Test foods were prepared by mixing a commercially available barium sulfate contrast medium (Baritop HD; Kaigen Pharma Co., Ltd., Osaka, Japan) with each vehicle. The test sequence followed institutional routine practice and included yogurt (International Dysphagia Diet Standardisation Initiative (IDDSI) level 4; semisolid), porridge (level 5), cooked rice (level 7), and thin liquid water (level 0) [28]. Fluoroscopic images were recorded continuously at 30 frames/s throughout the swallowing sequence and stored as digital video files for subsequent frame-by-frame analysis.

Because yogurt was the first semisolid bolus and was administered to all participants in one-tablespoon portions, quantitative analyses in this study focused on the first yogurt swallow for each patient. If a patient attempted the yogurt swallow more than once, only the first attempt was included in the analysis to avoid training or fatigue effects [3] and to minimize the influence of residue from prior attempts [29]. This approach was chosen to target a single standardized swallow condition and maximize between-patient comparability for both residue- and clearance-related quantitative metrics [15], given that airway invasion can vary by bolus volume and consistency [30]. For this reason, thin-liquid trials were performed as part of the clinical VFSS sequence but were not analyzed for the present quantitative comparison.

### 2.5. Image Analysis and Outcome Measures

Quantitative image analysis was performed using ImageJ software (version 1.54r; National Institutes of Health, Bethesda, MD, USA) following established protocols for VFSS research [15]. To ensure objectivity and minimize observer bias, all videofluoroscopic images were de-identified prior to analysis. A board-certified physiatrist with expertise in dysphagia, who was blinded to the patients’ clinical information and group allocation and was not involved in VFSS acquisition, performed the image analysis. To reduce measurement error and allow assessment of intra-rater reliability, all quantitative VFSS measures except the Penetration–Aspiration Scale (PAS) score were measured twice, and the mean value of the two measurements was used for the final statistical analysis. All images included in the video analysis were calibrated based on the diameter of a 10-won coin (22.86 mm) attached to the chin and neck of the patient to minimize measurement error.

**Figure 1:**
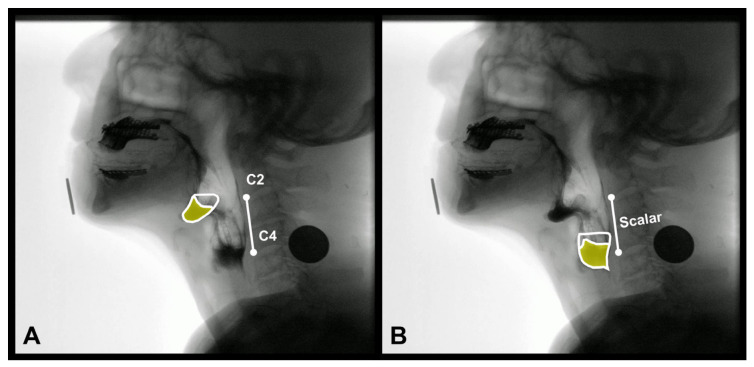
Calculation of the Normalized Residue Ratio Scale (NRRS). Representative post-swallow videofluoroscopic frames show region-of-interest tracings used to compute the NRRS in the valleculae (**A**) and piriform sinuses (**B**). The NRRS was calculated as (residue area/spatial housing area) × (residue area/(C2–C4)^2^) × 10, where spatial housing area denotes the area of the residue-housing compartment. The residue area (yellow shaded region) and spatial housing area (white outline) were traced within each compartment. “Scalar” denotes the C2–C4 distance used for anatomic normalization. Abbreviations and symbols: A = valleculae; B = piriform sinuses; C2, C4 = second and fourth cervical vertebral bodies; NRRS = Normalized Residue Ratio Scale; Scalar = C2–C4 distance.

The primary outcomes were post-swallow vallecular and piriform sinus residue quantified using the Normalized Residue Ratio Scale for the valleculae (NRRSv) and piriform sinuses (NRRSp) [15]. NRRS is a dimensionless, anatomically normalized residue measure that accounts for both compartment size (spatial housing area) and vertebral scaling. For NRRSv and NRRSp, residue area and the corresponding spatial housing area (i.e., the area of the residue-housing compartment) were traced as regions of interest for the valleculae and piriform sinuses, respectively, on the reference post-swallow frame. NRRS was calculated as (residue area/spatial housing area) × (residue area/(C2–C4)^2^) × 10, where C2–C4 (anatomic scalar) is the linear distance between the anterior-inferior corners of the C2 and C4 vertebral bodies (Figure 1). Higher NRRS values indicate greater pharyngeal residue.

**Figure 2:**
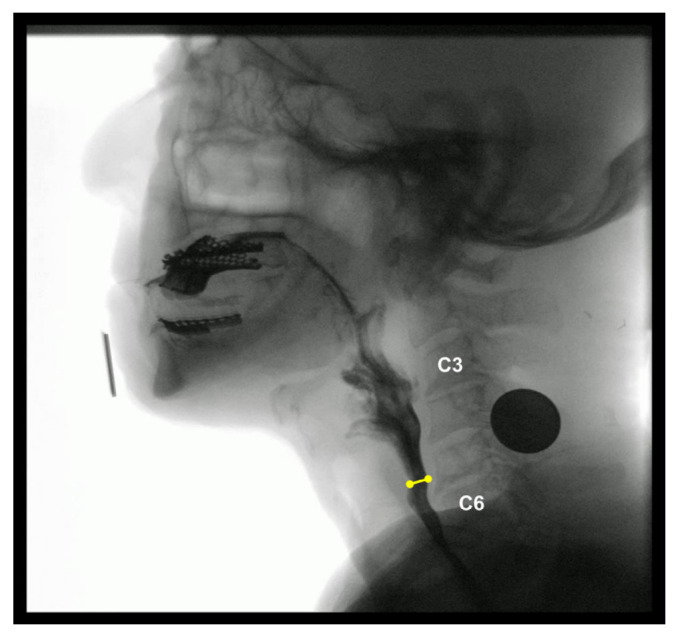
Upper esophageal sphincter (UES) opening width. Maximal UES opening width was measured on the videofluoroscopic frame demonstrating maximal UES opening during the standardized yogurt swallow. The yellow line segment indicates the maximal anteroposterior diameter (mm) at the narrowest pharyngoesophageal segment between the C3 and C6 vertebral levels. Abbreviations and symbols: C3, C6 = third and sixth cervical vertebral bodies; UES = upper esophageal sphincter.

UES opening width was defined as the maximal anteroposterior diameter (mm) at the narrowest pharyngoesophageal segment between the C3 and C6 vertebral levels during UES opening for the standardized yogurt swallow, measured on the frame demonstrating maximal UES opening (Figure 2) [31].

**Figure 3:**
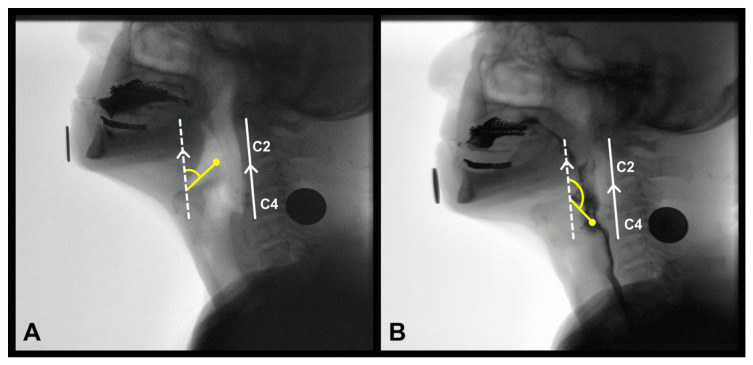
Epiglottic rotation angle. Representative videofluoroscopic frames show the pre-swallow position (**A**) and maximal epiglottic inversion (**B**). The epiglottic line (yellow) was drawn from the epiglottic base (root) to the epiglottic tip (yellow circle). The C2–C4 line (solid white line) was used as the reference axis; the dashed white line is a parallel reference line translated through the epiglottic base. White arrows indicate the parallel orientation of the reference lines, and the yellow arc indicates the measured angle. The epiglottic rotation angle was defined as the change in this angle from A to B (degrees); smaller values indicate more restricted epiglottic inversion. Abbreviations and symbols: A = pre-swallow frame; B = maximal inversion frame; C2, C4 = second and fourth cervical vertebral bodies.

Epiglottic inversion was quantified using an epiglottic rotation angle measure. The epiglottic line was drawn from the epiglottic base (root) to the epiglottic tip. The C2–C4 line (straight line connecting the anterior–inferior corners of the C2 and C4 vertebral bodies) served as the reference axis; to facilitate angle measurement, a line parallel to the C2–C4 reference axis was translated to pass through the epiglottic base. Epiglottic rotation angle was defined as the change in this angle from the pre-swallow frame to the frame of maximal inversion (degrees) (Figure 3) [32]. In this study, smaller epiglottic rotation angle values indicate more restricted epiglottic inversion.

**Figure 4:**
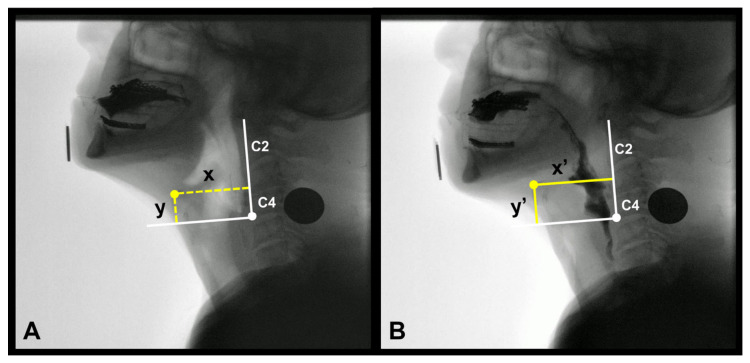
Hyoid displacement. Representative videofluoroscopic frames show the pre-swallow position (**A**) and maximal hyoid displacement (**B**). Hyoid position (yellow marker) was quantified in a C4-anchored coordinate system with the origin at the anterior-inferior corner of C4 (white marker). The *y*-axis was aligned with the C2–C4 line, and the *x*-axis was perpendicular to the *y*-axis (solid white lines). Coordinates were recorded as (x, y) in (**A**) (dashed yellow lines) and (x′, y′) in (**B**) (solid yellow lines). Anterior and vertical displacement were calculated as x′−x and y′−y, respectively, after correction for global image shift using the fixed C4 anchor point. Abbreviations and symbols: A = pre-swallow frame; B = maximal displacement frame; C2, C4 = second and fourth cervical vertebral bodies; x, y = hyoid coordinates at pre-swallow; x′, y′ = hyoid coordinates at maximal displacement.

Hyolaryngeal excursion was evaluated by measuring hyoid bone displacement in the anterior and vertical directions between the pre-swallow frame and the frame of maximal displacement [33]. The origin was set at the anterior–inferior corner of the C4 vertebral body; the y-axis was aligned with the C2–C4 line and the x-axis was defined as the line perpendicular to the y-axis. Hyoid coordinates were recorded as (x, y) on the pre-swallow frame and (x′, y′) on the frame of maximal displacement; anterior and vertical displacement components were computed as x′−x and y′−y, respectively, with correction for any global image shift using the fixed C4 anchor point (Figure 4) [34].

Pharyngeal transit time (PTT) was defined as the time interval in seconds from the moment the leading edge of the bolus passed the posterior nasal spine to the moment the bolus tail completely passed through the upper esophageal sphincter [35].

Airway invasion severity during the yogurt swallow was assessed using the Penetration–Aspiration Scale (PAS), an 8-point ordinal scale that reflects the depth of airway invasion and whether the material is expelled or remains in the airway [21]. For each participant, we recorded the PAS score for the first yogurt swallow. In addition, for descriptive analyses we categorized patients into three PAS severity groups: no airway invasion (PAS 1), penetration (PAS 2–5), and aspiration (PAS 6–8) [36].

### 2.6. Statistical Analysis

The primary outcomes of interest were the Normalized Residue Ratio Scale for the valleculae (NRRSv) and piriform sinuses (NRRSp) for the first standardized yogurt swallow, comparing the COVID-19 and AP groups. Prespecified secondary outcomes were UES opening width and epiglottic rotation angle, selected a priori based on their mechanistic relevance to pharyngeal residue. Exploratory outcomes included hyoid displacement, pharyngeal transit time, Penetration–Aspiration Scale (PAS) score, and the distribution of PAS severity groups.

Continuous variables were assessed for normality using the Shapiro–Wilk test and visual inspection of histograms and Q–Q plots. Normally distributed continuous variables are presented as means ± standard deviations (SD) and were compared between groups using the independent-samples Student’s *t*-test. Non-normally distributed continuous variables and ordinal variables are presented as medians with interquartile ranges (IQR) and were compared using the Mann–Whitney U test. Categorical variables are summarized as counts and percentages and were compared using Fisher’s exact test or the Pearson χ^2^ test, as appropriate; the 2 × 3 distribution of PAS severity groups was tested using the Pearson χ^2^ test.

For outcomes compared using the Mann–Whitney U test, we additionally reported the Hodges–Lehmann estimator (COVID–AP) with 95% confidence intervals (CIs) as a difference estimate. For outcomes compared using the Student’s *t*-test, we reported the mean difference (COVID–AP) with 95% CIs.

To address statistical power in this fixed retrospective cohort, we performed a retrospective simulation-based power estimate for the primary outcomes, NRRSv and NRRSp. Using the observed group-specific distributions, 10,000 resampled datasets were generat-ed with the original group sizes, and the proportion of two-sided Mann–Whitney U tests with *p* < 0.05 was calculated as the retrospective power estimate. This analysis was used only to contextualize the interpretability of the primary findings.

To address potential confounding by airway management, we performed a sensitivity analysis after excluding all patients with endotracheal intubation history. The same statistical framework as the main analysis was applied to the primary NRRS outcomes, key clearance-related secondary outcomes, and PAS score. Because of the reduced subgroup size, this analysis was interpreted descriptively.

Intra-rater reliability for the repeated quantitative VFSS measurements was assessed using intraclass correlation coefficients (ICCs) with 95% confidence intervals (CIs). Since the final analytic values were based on the mean of two measurements, a two-way mixed-effects, absolute-agreement, average-measures model was applied.

All statistical tests were two-sided, and a *p* value < 0.05 was considered statistically significant. Statistical analyses were conducted using R statistical software (version 4.2.2; R Foundation for Statistical Computing, Vienna, Austria). Given the modest sample size and multiple comparisons, no formal adjustment for multiplicity was performed; therefore, the results should be interpreted as exploratory and hypothesis-generating rather than confirmatory.

## 3. Results

### 3.1. Participant Flow and Baseline Characteristics

**Figure 5:**
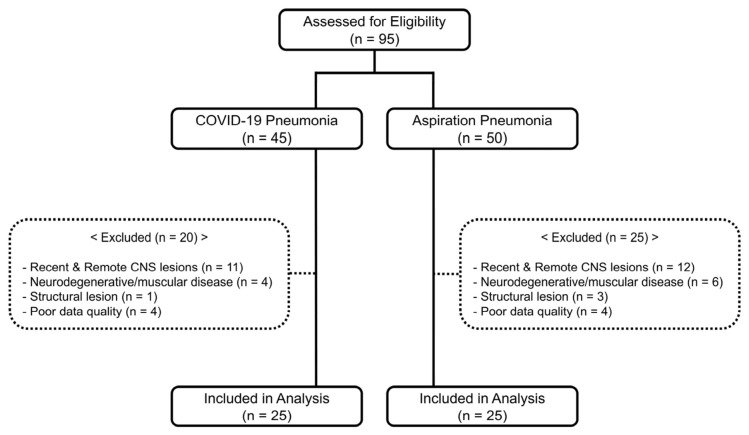
Flow diagram of patient selection. Flow diagram showing patient screening, exclusions, and final inclusion in the coronavirus disease 2019 (COVID-19) pneumonia and aspiration pneumonia (AP) cohorts for the study analysis. Abbreviations and symbols: COVID-19 = coronavirus disease 2019; AP = aspiration pneumonia; CNS = central nervous system; *n* = number of patients.

During the study period, 95 patients with pneumonia and dysphagia met the initial inclusion criteria (Figure 5). Of these, 45 had COVID-19 pneumonia and 50 had AP. After applying the exclusion criteria, 20 patients were excluded from the COVID-19 group and 25 from the AP group, yielding a final analytic sample of 25 patients in each group.

Baseline demographic and clinical characteristics are summarized in Table 1. The median age was 83.0 years [76.0–85.0] in the COVID-19 group and 80.0 years [66.0–84.0] in the AP group (*p* = 0.27). The proportion of male patients was 64.0% (16/25) in the COVID-19 group and 52.0% (13/25) in the AP group (*p* = 0.57).

No statistically significant between-group differences were detected in the clinical course and treatment intensity markers reported in Table 1. Total hospital days did not differ significantly between groups (21.0 [12.0–32.0] vs. 16.0 [7.0–28.0] days, *p* = 0.34), nor did total ICU days (10.0 [5.0–16.0] vs. 2.0 [0.0–14.0] days, *p* = 0.14). The time interval from pneumonia diagnosis to VFSS was 19.0 [13.0–37.0] days in the COVID-19 group and 16.0 [7.0–28.0] days in the AP group (*p* = 0.08). Airway management variables were also similar, including history of endotracheal intubation (20.0% vs. 40.0%, *p* = 0.22) and tracheostomy (8.0% vs. 12.0%, *p* > 0.99). Among patients who underwent these procedures, cumulative endotracheal intubation duration (*p* = 0.33) and cumulative tracheostomy duration (*p* = 0.20) during the interval between pneumonia diagnosis and VFSS were also comparable. Overall, none of the baseline variables listed in Table 1 showed a statistically significant between-group difference. Although median ICU days were numerically longer in the COVID-19 group, the wide and overlapping IQRs indicate substantial clinical heterogeneity in treatment intensity within both groups. Therefore, ICU days should be interpreted as a descriptive marker of clinical courses rather than as a precise severity-matching variable.

### 3.2. Quantitative Swallowing Outcomes

Quantitative VFSS outcomes for the first standardized yogurt swallow are shown in Table 2. Regarding the primary outcomes, NRRSv was significantly higher in the COVID-19 group compared with the AP group (Hodges–Lehmann estimator 0.12 [95% CI 0.01 to 0.25]; *p* = 0.01). NRRSp was numerically higher in the COVID-19 group but did not reach statistical significance (Hodges–Lehmann estimator 0.05 [95% CI 0.00 to 0.22]; *p* = 0.07).

Retrospective simulation-based power estimates were 72.0% for NRRSv and 44.6% for NRRSp. These estimates indicate moderate power for detecting the observed be-tween-group NRRSv difference but limited power for NRRSp. Therefore, the non-significant NRRSp result should be interpreted as statistically inconclusive rather than as evidence of equivalence between groups. These results are summarized in Supplementary Table S1.

For the prespecified secondary outcomes, UES opening width was significantly smaller in the COVID-19 group (mean difference -1.42 mm [95% CI −2.71 to −0.14]; *p* = 0.03). Epiglottic inversion, quantified by the epiglottic rotation angle, was also significantly more restricted in the COVID-19 group (Hodges–Lehmann estimator −22.1° [95% CI −45.1 to −1.4]; *p* = 0.04).

**Figure 6:**
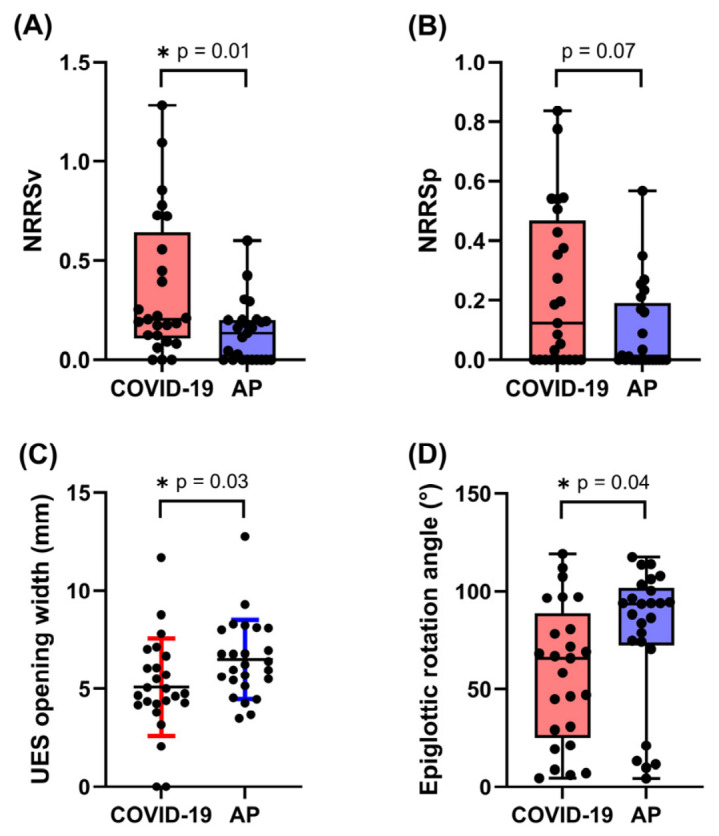
Summary of quantitative videofluoroscopic swallowing study (VFSS) measures from the first standardized semisolid yogurt swallow in the coronavirus disease 2019 (COVID-19) pneumonia and clinically diagnosed aspiration pneumonia (AP) groups. Individual patient data are superimposed as scatter points (*n* = 25 per group). (**A**) Normalized Residue Ratio Scale for the valleculae (NRRSv), (**B**) Normalized Residue Ratio Scale for the piriform sinuses (NRRSp), and (**D**) epiglottic rotation angle are non-normally distributed variables; data are presented as box-and-whisker plots indicating the median (horizontal line), interquartile range (box ends), and minimum–maximum values (whiskers), with *p*-values calculated using the Mann–Whitney U test. (**C**) Upper esophageal sphincter (UES) opening width is a normally distributed variable; data are presented with central horizontal lines and error bars indicating the mean ± standard deviation, with the *p*-value calculated using the independent-samples Student’s *t*-test. Asterisks (*) indicate statistical significance (*p* < 0.05). Abbreviations: AP = aspiration pneumonia; COVID-19 = coronavirus disease 2019; NRRSp = Normalized Residue Ratio Scale for the piriform sinuses; NRRSv = Normalized Residue Ratio Scale for the valleculae; UES = upper esophageal sphincter; VFSS = videofluoroscopic swallowing study.

Figure 6 visually summarizes the between-group distributions of the primary residue outcomes (Figure 6A,B) and prespecified clearance-related secondary outcomes (Figure 6C,D) for the first standardized semisolid yogurt swallow; detailed estimates and confidence intervals are reported in Table 2.

Exploratory measures showed no significant between-group differences, including hyoid anterior displacement (*p* = 0.24), hyoid vertical displacement (*p* = 0.42), and pharyngeal transit time (*p* = 0.11). The ordinal PAS score on the yogurt swallow did not differ significantly between groups (*p* = 0.85). Detailed difference estimates and confidence intervals for all outcomes are provided in Table 2. The repeated quantitative VFSS measurements demonstrated excellent intra-rater reliability across all variables (average-measures ICC range: 0.994–0.999; 95% CI lower bounds ≥ 0.990).

In the sensitivity analysis excluding patients with endotracheal intubation history, 20 patients in the COVID-19 group and 15 patients in the AP group were included. The overall direction of the residue- and clearance-related estimates remained generally consistent with the main analysis, although statistical significance was attenuated for some outcomes. The COVID-19 group continued to show a higher median NRRSv than the AP group (0.18 [0.09–0.31] vs. 0.11 [0.00–0.18]; Hodges–Lehmann difference 0.08, 95% CI −0.01 to 0.19; *p* = 0.096). UES opening width remained significantly smaller in the COVID-19 group (4.88 ± 2.56 vs. 6.62 ± 2.27 mm; mean difference −1.74 mm, 95% CI −3.43 to −0.04; *p* = 0.045). Epiglottic rotation angle also remained directionally lower in the COVID-19 group (67.58 [27.07–84.75] vs. 93.42 [74.43–104.96] degrees; Hodges–Lehmann difference −20.70 degrees, 95% CI −48.39 to 3.19; *p* = 0.092). NRRSp and PAS score showed no statistically significant between-group differences in this reduced subgroup. These descriptive sensitivity findings are presented in Supplementary Table S1.

### 3.3. Airway Invasion Severity Outcomes

PAS severity group distributions for the yogurt swallow are summarized in Table 3. The proportion of patients with no airway invasion (PAS 1) was 40.0% (10/25) in the COVID-19 group and 48.0% (12/25) in the AP group. Penetration (PAS 2–5) was observed in 40.0% (10/25) of the COVID-19 group and 20.0% (5/25) of the AP group, while aspiration (PAS 6–8) occurred in 20.0% (5/25) and 32.0% (8/25), respectively. The overall distribution of PAS severity groups did not differ significantly between groups (*p* = 0.28), indicating that no statistically significant difference in airway invasion severity distribution was detected on the standardized semisolid task.

## 4. Discussion

In this retrospective cohort study, we compared quantitative VFSS measures between hospitalized patients with COVID-19 pneumonia and those with clinically diagnosed aspiration pneumonia (AP) under a standardized semisolid yogurt condition. To our knowledge, this is the first VFSS study to apply image-based quantitative residue measures and clearance-related kinematics to compare swallowing biomechanics between patients with COVID-19 pneumonia and clinically diagnosed aspiration pneumonia. Consistent with our prespecified primary hypothesis, the COVID-19 group demonstrated significantly greater post-swallow vallecular residue (higher NRRSv). While piriform sinus residue (NRRSp) was numerically higher in the COVID-19 group, this difference did not reach statistical significance. Secondary measures further characterized this phenotype, showing significantly smaller UES opening width and a more restricted epiglottic rotation angle in the COVID-19 group. In contrast, exploratory measures—specifically hyoid anterior and vertical displacement, pharyngeal transit time, and PAS-based airway invasion—did not differ between groups. Importantly, these biomechanical differences were observed even though no statistically significant between-group differences were detected in the measured clinical course variables, including cumulative intubation and tracheostomy days, ICU days, and time from pneumonia diagnosis to VFSS. Although these coarse markers do not exclude unmeasured confounding, the observed phenotype differences suggest a distinct efficiency-predominant pattern in COVID-19 and should be interpreted as exploratory and hypothesis-generating signals.

### 4.1. The COVID-19 Dysphagia Phenotype: Pharyngeal Residue and Biomechanical Constraints

The COVID-19 pneumonia group exhibited a dysphagia profile characterized by reduced pharyngeal clearance efficiency compared with the AP group. This was primarily driven by a significantly higher burden of vallecular residue (NRRSv). Piriform sinus residue (NRRSp) was numerically higher in the COVID-19 group, and although this difference did not reach statistical significance (*p* = 0.07), this pattern is directionally consistent with the restricted UES opening width observed in the COVID-19 group and warrants confirmation in larger samples. Collectively, these findings suggest that patients with COVID-19 pneumonia faced greater challenges in effective pharyngeal clearance relative to the AP cohort.

These clearance-related findings were accompanied by smaller UES opening width [37] and a reduced epiglottic rotation angle [20], two VFSS-derived kinematic measures plausibly related to pharyngeal residue. Epiglottic inversion is generally considered a largely passive event influenced by hyolaryngeal excursion and, potentially, bolus-structure interactions [38]. In our data, epiglottic rotation angle was reduced even though hyoid displacement did not differ significantly between groups, suggesting that factors beyond gross hyoid excursion may contribute [39]. One hypothesis is reduced pharyngolaryngeal compliance (tissue stiffness), which cannot be directly assessed with our kinematic measures. UES opening width serves as a VFSS-derived kinematic surrogate for sphincter opening magnitude [40]; a narrower maximal opening could increase resistance to bolus outflow [17]. Given that efficient pharyngeal clearance depends on both adequate pharyngeal driving forces [41] and an open outflow tract (UES) [40], the observed reductions in epiglottic rotation angle and UES opening width may contribute to increased upstream residue.

The observed biomechanical pattern—reduced UES opening and restricted epiglottic inversion—is theoretically compatible with the pathophysiology of COVID-19-associated acute sarcopenia [42] or critical illness myopathy [10]. Systemic inflammation and viral-mediated muscle wasting [43] might disproportionately compromise the swallowing musculature in patients with COVID-19. Furthermore, potential neurotropic effects of SARS-CoV-2 involving the pharyngeal sensorimotor plexus have been proposed, as viral entry sites identified in the vagus and glossopharyngeal nerves [11] could hypothetically disrupt the precise sensorimotor coordination required for both epiglottic inversion and UES opening. However, because the present study did not directly assess objective measures of muscle mass or detailed neurologic status, these mechanisms should be regarded as plausible hypotheses rather than causal explanations of the VFSS phenotype observed in this cohort. In contrast, pharyngeal transit time did not differ significantly between groups. Because PTT reflects transit duration and we did not quantify swallow initiation delay or stage transition measures, timing-related abnormalities cannot be excluded based solely on these data.

### 4.2. Dissociation Between Swallowing Efficiency and Safety

Our findings highlight a marked dissociation between swallowing efficiency (residue) and airway safety (penetration–aspiration) in patients with COVID-19. Despite harboring a significantly greater burden of pharyngeal residue and exhibiting distinct biomechanical restrictions, the COVID-19 group did not differ from the AP group in PAS scores or the occurrence of aspiration on the standardized semisolid task.

This dissociation suggests a ‘latent aspiration risk,’ where substantial post-swallow residue increases the potential for postdeglutitive aspiration [44] despite comparable PAS scores during the swallow. The cohesive nature of the semisolid bolus likely minimized immediate airway entry, whereas thin liquids might more readily provoke invasion [30]. However, the accumulated residue serves as a reservoir for potential aspiration after the recorded sequence [44], given that PAS captures events only within the brief fluoroscopic window. Thus, the lack of a statistically significant difference in immediate airway invasion on a semisolid trial implies a specific efficiency deficit rather than preserved safety; delayed post-swallow aspiration events were not directly monitored in this study.

### 4.3. Phenotypic Heterogeneity in Aspiration Pneumonia

The observation of comparatively lower pharyngeal residue in the clinically diagnosed AP group warrants careful interpretation. This finding likely reflects the inherent phenotypic heterogeneity of aspiration pneumonia as a clinical syndrome [7], rather than superior swallowing physiology in this population. Unlike the COVID-19 cohort, which shared a specific viral etiology and acute pathophysiologic mechanism, the AP group comprised patients with diverse underlying causes, often involving multifactorial etiologies associated with aging and frailty [6].

In such heterogeneous AP populations [6], delayed swallowing reflexes or sensory impairment may contribute to aspiration risk [45]. However, because the present quantitative comparison was limited to the first standardized semisolid yogurt swallow, we could not fully capture the safety-predominant profile typically associated with general AP, which is often more evident with thin liquids [30]. Nonetheless, under identical semisolid conditions, the AP group did not exhibit the severe residue-predominant clearance impairment observed in the COVID-19 pneumonia group. Therefore, the significant between-group clearance-related differences—higher vallecular residue, restricted epiglottic inversion, and reduced UES opening in the COVID-19 pneumonia group—strongly support a distinct efficiency-predominant dysphagia phenotype. Accordingly, comparatively lower semisolid residue in the AP group should not be interpreted as superior overall swallowing safety; because airway invasion can vary by bolus viscosity and consistency [28], airway-safety interpretations across other consistencies, particularly thin liquids, should be made cautiously.

### 4.4. Clinical Implications

The identification of this efficiency-impaired phenotype has direct implications for the management of dysphagia in patients with COVID-19. First, regarding diagnosis, our findings indicate a dissociation between swallowing safety and efficiency. Although PAS scores were comparable to the AP group, the COVID-19 group exhibited significant vallecular residue and biomechanical restrictions, specifically reduced UES opening and epiglottic inversion. Clinically, these findings suggest that dysphagia assessment in patients with COVID-19 pneumonia should not rely solely on PAS-defined airway invasion. An apparently safe swallow on semisolid consistency does not preclude the presence of significant residue [30], which represents a reservoir for delayed post-swallow aspiration [46] once the patient resumes respiration or changes posture. Therefore, early instrumental evaluation in high-risk individuals [47] and careful quantification of pharyngeal clearance [15] may help inform the selection of clearance-focused compensatory strategies and staged rehabilitation, even when overt coughing or immediate aspiration is not observed under that semisolid condition [48].

From a rehabilitation perspective, the specific biomechanical deficits identified—restricted UES opening and epiglottic inversion—theoretically support interventions targeting hyolaryngeal excursion and sphincter opening, such as the Shaker exercise [49] or the Mendelsohn maneuver [50]. However, application of these intensive maneuvers requires caution, given that patients with severe COVID-19 pneumonia often suffer from respiratory compromise, hypoxemia, and fatigue [51]. Accordingly, a phased management approach tailored to patient tolerance may be considered. In the early recovery phase, maximizing clearance through compensatory strategies, such as cyclic ingestion [52], multiple dry swallows [53], and effortful swallows [54], along with texture modification using standardized terminology (e.g., IDDSI) [28], may be prioritized to manage residue burden without overwhelming the patient’s respiratory reserve. As systemic condition and respiratory endurance improve, a transition to active restorative exercises targeting the suprahyoid musculature and UES function can be implemented to address the underlying biomechanical restrictions [49].

### 4.5. Limitations and Future Directions

This study has several limitations. First, the retrospective single-center design and modest sample size limit causal inference and statistical power, particularly for NRRSp and exploratory outcomes. Accordingly, non-significant findings should be interpreted as the absence of statistically detectable differences rather than evidence of true equivalence. Although we observed distinct phenotypic patterns, potential selection bias exists because the inclusion criteria required patients to maintain a sitting position for VFSS [26], which likely excluded patients with severe physical deconditioning or lower levels of consciousness. Furthermore, unmeasured residual confounding, such as baseline frailty, may have influenced the results [55]. Although the sensitivity analysis excluding patients with endotracheal intubation history showed a generally consistent direction for the primary vallecular residue and clearance-related kinematic findings (Supplementary Table S1), the reduced subgroup size limited statistical precision. Therefore, residual confounding related to airway management, illness severity, and post-intubation laryngopharyngeal dysfunction cannot be fully excluded in this retrospective cohort. In addition, since aspiration pneumonia was diagnosed clinically and is inherently heterogeneous [7], etiologic misclassification may have attenuated group-level signals in the AP cohort [56].

Second, our quantitative analysis was restricted to the first standardized semisolid bolus. Although this approach maximized between-group comparability and minimized fatigue, the present findings should not be generalized to thin-liquid swallowing or other bolus consistencies. Thin-liquid water was included in the clinical VFSS sequence but was not analyzed quantitatively in this study. Therefore, consistency-dependent airway invasion patterns, particularly thin-liquid penetration or aspiration, require future multi-consistency analyses. In addition, our pharyngeal timing metric (PTT) focused on transit duration and did not capture swallow initiation delay or stage transition measures. Moreover, airway safety was assessed only within the brief fluoroscopic window, potentially underestimating the risk of delayed aspiration from residue [46] after the patient resumes respiration or continues oral intake.

Third, mechanistic interpretation is limited by the absence of direct muscle mass measurements, formal neurologic assessment, and high-resolution pharyngeal manometry [57]. Although UES opening width is a VFSS-derived kinematic measure, it does not directly quantify intrabolus pressure generation or sphincter relaxation. Future prospective studies should incorporate multi-consistency VFSS protocols, high-resolution pharyngeal manometry or impedance manometry, objective measures of muscle mass and frailty, formal neurologic assessments, and longitudinal clinical outcomes such as pneumonia recurrence, tube-feeding duration, and recovery of oral intake.

## 5. Conclusions

In conclusion, this study suggests a distinct efficiency-predominant dysphagia phenotype in patients with COVID-19 pneumonia compared with those with clinically diagnosed aspiration pneumonia. This phenotype is characterized by significantly greater vallecular residue and concordant restrictive pharyngeal mechanics—specifically, reduced UES opening and restricted epiglottic inversion. Notably, these biomechanical deficits occurred despite no statistically significant between-group difference in airway safety scores on the standardized semisolid task. This pattern suggests a clinical dissociation between swallowing safety and efficiency that may mask latent aspiration risk. While these findings highlight a distinct biomechanical profile in COVID-19 pneumonia, prospective studies using multi-consistency protocols and physiologic measures such as high-resolution pharyngeal manometry, and evaluating longitudinal clinical outcomes are warranted to further validate this phenotype and clarify its clinical significance.

## Figures and Tables

**Table 1:** Baseline characteristics of the study cohort.

Variables	COVID-19 (*n* = 25)	AP (*n* = 25)	*p*-Value
Age, yr	83.0 [76.0–85.0]	80.0 [66.0–84.0]	0.27
Sex			0.57
Male	16 (64.0)	13 (52.0)	
Female	9 (36.0)	12 (48.0)	
Clinical course			
Total hospital days	21.0 [12.0–32.0]	16.0 [7.0–28.0]	0.34
Total ICU days	10.0 [5.0–16.0]	2.0 [0.0–14.0]	0.14
Time from pneumonia diagnosis to VFSS, days	19.0 [13.0–37.0]	16.0 [7.0–28.0]	0.08
Airway management			
Endotracheal intubation history	5 (20.0)	10 (40.0)	0.22
Endotracheal intubation duration, days *	20.0 [18.0–22.0] (*n* = 5)	15.5 [7.8–20.8] (*n* = 10)	0.33
Tracheostomy history	2 (8.0)	3 (12.0)	>0.99
Tracheostomy duration, days *	29.5 [25.2–33.8] (*n* = 2)	16.0 [13.0–18.0] (*n* = 3)	0.20

Note: COVID-19 = coronavirus disease 2019; AP = aspiration pneumonia; *n* = number of patients; yr = year; ICU = intensive care unit; VFSS = videofluoroscopic swallowing study. Values are presented as median [interquartile range] or number (%). *p*-values were calculated using the Mann–Whitney U test for continuous variables and Fisher’s exact test for categorical variables. * Cumulative durations (days) of endotracheal intubation and tracheostomy during the interval between pneumonia diagnosis and VFSS are summarized among patients who underwent each procedure; subgroup *n* is shown in parentheses.

**Table 2:** Comparison of quantitative videofluoroscopic swallowing study outcome measures between the coronavirus disease 2019 pneumonia and aspiration pneumonia groups.

Outcomes	COVID-19 (*n* = 25)	AP (*n* = 25)	Difference (95% CI)	*p*-Value
Primary Outcomes				
NRRSv	0.20 [0.12–0.56]	0.13 [0.00–0.20]	0.12 [0.01, 0.25]	0.01 *
NRRSp	0.12 [0.00–0.43]	0.00 [0.00–0.17]	0.05 [0.00, 0.22]	0.07
Secondary Outcomes				
UES opening width, mm	5.08 ± 2.48	6.50 ± 2.01	−1.42 [−2.71, −0.14]	0.03 *
Epiglottic rotation angle, °	66.0 [29.0–80.8]	93.4 [74.2–100.4]	−22.1 [−45.1, −1.4]	0.04 *
Exploratory Outcomes				
Hyoid anterior displacement (mm)	3.69 [2.19–8.09]	6.40 [3.05–7.39]	−1.21 [−3.35, 0.95]	0.24
Hyoid vertical displacement (mm)	12.90 [6.24–17.09]	9.76 [3.93–16.22]	1.83 [−2.92, 6.08]	0.42
Pharyngeal transit time, s	2.31 [1.66–4.54]	1.76 [1.25–4.15]	0.49 [−0.13, 1.38]	0.11
PAS score	2 [1–5]	2 [1–7]	0.00 [−1.00, 1.00]	0.85

Note: COVID-19 = coronavirus disease 2019; AP = aspiration pneumonia; *n* = number of patients; CI = confidence interval; NRRSv = Normalized Residue Ratio Scale for the valleculae; NRRSp = Normalized Residue Ratio Scale for the piriform sinuses; UES = upper esophageal sphincter; mm = millimeter; s = second; ° = degree; PAS = Penetration–Aspiration Scale. Values are presented as median [interquartile range] or mean ± standard deviation. *p*-values were calculated using the Mann–Whitney U test for non-normally distributed variables and the independent-samples Student’s *t*-test for normally distributed variables. Differences are reported as the Hodges–Lehmann estimator (COVID-19–AP) with 95% confidence intervals for variables summarized as median [interquartile range] and as the mean difference (COVID-19–AP) with 95% confidence intervals for variables summarized as mean ± standard deviation. * Indicates statistical significance (*p* < 0.05).

**Table 3:** Comparison of airway invasion severity distribution between the coronavirus disease 2019 pneumonia and aspiration pneumonia groups.

Outcomes	COVID-19 (*n* = 25)	AP (*n* = 25)	*p*-Value
PAS severity group, *n* (%)			0.28 †
No airway invasion (PAS 1)	10 (40.0)	12 (48.0)	
Penetration (PAS 2–5)	10 (40.0)	5 (20.0)	
Aspiration (PAS 6–8)	5 (20.0)	8 (32.0)	

Note: COVID-19 = coronavirus disease 2019; AP = aspiration pneumonia; *n* = number of patients; PAS = Penetration–Aspiration Scale. Values are presented as number (%). † *p*-value was calculated using the Pearson χ^2^ test (2 × 3).

## Data Availability

The data presented in this study are available from the corresponding author upon reasonable request. The data are not publicly available due to privacy and ethical restrictions related to patient-level clinical and videofluoroscopic swallowing study data.

## Supplementary Materials

The following supporting information can be downloaded at https://www.mdpi.com/article/10.3390/medicina62071212/s1, Table S1: Retrospective power estimates and sensitivity analysis excluding patients with endotracheal intubation history.

## Abbreviations

The following abbreviations are used in this manuscript:

AP	aspiration pneumonia
CI	confidence interval
CNS	central nervous system
COVID-19	coronavirus disease 2019
ICC	intraclass correlation coefficient
ICU	intensive care unit
IDDSI	international dysphagia diet standardisation initiative
IQR	interquartile range
IRB	institutional review board
NRRS	normalized residue ratio scale
NRRSp	normalized residue ratio scale for the piriform sinuses
NRRSv	normalized residue ratio scale for the valleculae
PAS	penetration–aspiration scale
PTT	pharyngeal transit time
RT-PCR	real-time reverse-transcription polymerase chain reaction
SARS-CoV-2	severe acute respiratory syndrome coronavirus 2
SD	standard deviation
UES	upper esophageal sphincter
VFSS	videofluoroscopic swallowing study
