# Supplementary material for: Dysphagia Phenotypes in COVID-19 Pneumonia Versus Aspiration Pneumonia: A Retrospective Quantitative Videofluoroscopic Cohort Study
Source: Medicina (Kaunas). 2026 Jun 23;62(7):1212. doi: 10.3390/medicina62071212 (PMC13414394; doi:10.3390/medicina62071212)
Supplement: Supplementary file 1 [file medicina-62-01212-s001.zip › medicina-4360377-supplementary.pdf]

Table S1. Retrospective power estimates and sensitivity analysis excluding patients with endotracheal intubation history

Panel A. Retrospective simulation-based power estimates for primary NRRS outcomes

Outcome	COVID-19 (n = 25)	AP (n = 25)	Observed p-Value	Retrospective power estimate	MC 95% CI
NRRSv	0.20 [0.12–0.56]	0.13 [0.00–0.20]	0.014	72.0%	71.1–72.9%
NRRSp	0.12 [0.00–0.43]	0.00 [0.00–0.17]	0.073	44.6%	43.6–45.6%

Panel B. Sensitivity analysis after excluding patients with endotracheal intubation history

Outcome	COVID-19 non-intubated (n = 20)	AP non-intubated (n = 15)	Difference (95% CI)	p-Value
NRRSv	0.18 [0.09–0.31]	0.11 [0.00–0.18]	0.08 [−0.01, 0.19]	0.096
NRRSp	0.04 [0.00–0.29]	0.00 [0.00–0.19]	0.00 [0.00, 0.12]	0.333
UES opening width, mm	4.88 ± 2.56	6.62 ± 2.27	−1.74 [−3.43, −0.04]	0.045
Epiglottic rotation angle, °	67.58 [27.07–84.75]	93.42 [74.43–104.96]	−20.70 [−48.39, 3.19]	0.092
PAS score	2.00 [1.00–3.50]	2.00 [1.00–2.50]	0.00 [−1.00, 1.00]	0.599

PAS severity group distribution in the non-intubated sensitivity analysis

PAS severity group	COVID-19 non-intubated (n = 20)	AP non-intubated (n = 15)	p-Value
Overall distribution			0.909
Normal (PAS 1), n (%)	8 (40.0)	7 (46.7)	
Penetration (PAS 2–5), n (%)	8 (40.0)	5 (33.3)	
Aspiration (PAS 6–8), n (%)	4 (20.0)	3 (20.0)	

Notes: Panel A: Retrospective power estimates were calculated from 10,000 resampled datasets generated from the observed group-specific empirical distributions with the original group sizes. Statistical significance was defined as a two-sided Mann–Whitney U test p-value < 0.05. MC 95% CI denotes the Monte Carlo confidence interval for the simulation proportion. NRRSv showed moderate simulation-based power for the observed between-group difference, whereas NRRSp showed limited power; therefore, the non-significant NRRSp result should be interpreted as statistically inconclusive rather than evidence of equivalence. Panel B: Values are presented as median [IQR], mean ± SD, or number (%). Difference estimates are Hodges–Lehmann estimators (COVID-19–AP) with 95% CIs for Mann–Whitney U outcomes and mean differences (COVID-19–AP) with 95% CIs for Student t-test outcomes. p-values were calculated using Mann–Whitney U tests for NRRSv, NRRSp, epiglottic rotation angle, and PAS score; Student t-test for UES opening width; and Pearson χ^2 test for the PAS severity group distribution. The sensitivity analysis was interpreted descriptively because of the reduced subgroup size.

Abbreviations: AP = aspiration pneumonia; CI = confidence interval; IQR = interquartile range; MC = Monte Carlo; NRRSv = Normalized Residue Ratio Scale for the valleculae; NRRSp = Normalized Residue Ratio Scale for the piriform sinuses; PAS = Penetration–Aspiration Scale; SD = standard deviation; UES = upper esophageal sphincter.
